# Disproportionate reduction in respiratory vs. non-respiratory outpatient clinic visits and antibiotic use in children during the COVID-19 pandemic

**DOI:** 10.1186/s12887-022-03315-0

**Published:** 2022-05-06

**Authors:** Noga Givon-Lavi, Dana Danino, Bart Adriaan van der Beek, Amir Sharf, David Greenberg, Shalom Ben-Shimol

**Affiliations:** 1 Faculty of Health Sciences, Ben-Gurion University of the Negev, Beer-Sheva, Israel; 2 The Pediatric Infectious Disease Unit, Soroka University Medical Center, Beer-Sheva, Israel; 3 Economics and Data Analysis Department, Clalit HMO South District, Beer-Sheva, Israel

**Keywords:** COVID-19, Pediatric outpatient clinic visits, Antibiotic use

## Abstract

**Background:**

The COVID-19 pandemic led to improved hygiene and reduced social encounters. Near elimination of the activity of respiratory syncytial virus and influenza viruses were observed, worldwide. Therefore, we assessed the rates of pediatric outpatient clinic visits and medications prescribed at those visits during the coronavirus disease 2019 (COVID-19) pandemic and pre-COVID-19 period (2016–2019).

**Methods:**

Monthly and annual incidence rates for respiratory and non-respiratory diagnoses and dispensed prescription rates were calculated. Acute gastroenteritis (AGE) visits were analyzed separately since the mode of transmission is influenced by hygiene and social distancing.

**Results:**

Overall, 5,588,702 visits were recorded. Respiratory and AGE visits declined by 49.9% and 47.3% comparing the COVID-19 and pre-COVID-19 periods. The respective rate reductions for urinary tract infections, trauma, and skin and soft tissue infections were 18.2%, 19.9%, and 21.8%. Epilepsy visits increased by 8.2%. Overall visits rates declined by 21.6%. Dispensed prescription rates of antibiotics and non-antibiotics respiratory medications declined by 49.3% and 44.4%, respectively. The respective declines for non-respiratory antibiotics and non-antibiotics were 15.1% and 0.2%. Clinic visits and prescription rates reductions were highest in April–May, following the first lockdown in Israel.

**Conclusions:**

COVID-19 pandemic resulted in a substantial reduction in respiratory outpatient clinic visits and dispensed respiratory drugs, with only a mild reduction seen for non-respiratory visits. These trends were probably driven by COVID-19 mitigation measures and by the profound disruption to non-SARS COV-2 respiratory virus activity.

**Supplementary Information:**

The online version contains supplementary material available at 10.1186/s12887-022-03315-0.

## Background

Most pediatric coronavirus disease 2019 (COVID-19) cases are milder compared with COVID-19 in adults, and only a minority of children with SARS-CoV-2 infection require hospitalization [1, 2]. However, the COVID-19 pandemic resulted in unexpected changes in pediatric healthcare service utilization.

The two main processes that determined pediatric outpatient clinic use during the COVID-19 pandemic were changes in human behavior and SARS-CoV-2’s impact on respiratory illness rates. In terms of human behavior, factors that affected pediatric outpatient clinic utilization included public fear from being exposed to the healthcare environment [3] and additionally, COVID-19 mitigation measures that led to improved hygiene and reduced social encounters (i.e., social distancing). With regard to the impact of SARS-CoV-2 on clinic visits, this was driven by a global change in the typical seasonal pattern of other respiratory viruses, most notably the near elimination of the activity of respiratory syncytial virus (RSV) and influenza viruses [4–8]. Indeed, multiple worldwide reports have pointed to reduced pediatric clinic visits and hospitalizations, mainly for respiratory diseases [9–13]. In addition, early reports on antibiotic prescription rates during the first months of the COVID-19 pandemic showed a decline in antibiotic use beyond the expected seasonal decline, as compared to the same period in previous years [14, 15].

The first case of COVID-19 in Israel was identified on February 21^st^, 2020 (in the middle of the respiratory season). Two weeks later, wide-scale social distancing measures were implemented by the Israeli Ministry of Health (MOH), and a complete nationwide lockdown came into effect in April 2020 [16].

In southern Israel ~ 80% of the pediatric population receive medical services from the same health maintenance organization (HMO), in which all visits and dispensed drug prescriptions are computerized.

Since both human behavioral changes and SARS-CoV-2’s impact on respiratory illnesses are expected to mostly impact respiratory diseases, we hypothesized that COVID-19 pandemic would have a greater impact on the rates of respiratory visits than on those of non-respiratory visits. Therefore, our aim was to compare the COVID-19 pandemic period rates to the pre-COVID-19 period (2016–2019) rates of pediatric outpatient clinic respiratory visits vs. non-respiratory visits in association with drugs prescribed for respiratory diagnoses versus drugs prescribed for non-respiratory diagnoses.

## Methods

### Study population and design

In 2016 and 2020 ~ 280,000 and ~ 310,000 children < 18 years old, respectively, inhabited the Negev region of southern Israel [17, 18]. In this region, two distinct ethnic groups, the Jewish and the Bedouin populations live side by side. The Jewish population is largely urban, whereas the Bedouin population, formally desert nomads, is in transition to a Western lifestyle. Among the Bedouin children, crowding, high pneumococcal carriage rates and hospitalization for respiratory infections were more prevalent [19]. In the pre-pneumococcal conjugate vaccine era, higher rates of dispensed antibiotics were observed among young Bedouin children compared to Jewish children [19]. However, following the implementation of pneumococcal conjugate vaccines, reductions in antibiotic use were seen in both populations but were more accentuated among Bedouin children in any given age group and with most drugs, resulting in almost complete closure of the gap between the two ethnic groups [20].

In 2016 and 2020, ~ 80% of all children < 18 years old received medical services by the Clalit HMO. All Clalit HMO clinics with ≥ 50 insured children < 18 years old, active throughout the study period, were included.

The study was approved by the Ethics Committee of Soroka University Medical Center.

Monthly overall clinic visits, visit diagnoses and dispensed medication rates (per 1,000 insured children) were recorded and divided into two main categories: respiratory and non-respiratory. Visits diagnoses categories were divided into respiratory visits, acute gastroenteritis (AGE) and non-respiratory disease, non-AGE visits. Respiratory visits included upper respiratory tract infections (URI), acute otitis media (AOM), lower respiratory tract infections (LRI) and asthma. AGE visits were analyzed separately, since we expected AGE rates to decline due to AGE mode of transmission (fecal–oral) is affected by hand hygiene, social distancing and school closure. Non-respiratory, non-AGE visits included skin and soft tissue infections (SSTI), urinary tract infections (UTI), epilepsy and trauma, which were the four most common non-respiratory, non-AGE diagnoses and were chosen as controls.

Dispensed medication prescriptions were divided into respiratory drugs and non-respiratory drugs. Respiratory drugs included respiratory antibiotics: amoxicillin/amoxicillin-clavulanate (amoxicillin and amoxicillin-clavulanate were consolidated into a single category since they are the most commonly used antibiotics for respiratory tract infections) [21, 22], azithromycin and ceftriaxone, and respiratory non-antibiotics drugs: asthma inhalators and solutions, sore throat relief and nasal anti-congestion drugs. Non-respiratory drugs included non-respiratory antibiotics: oral first generation cephalosporins and trimethoprim/sulfamethoxazole, and non-respiratory, non-antibiotic drugs: anti-epileptic drugs. Of note, antibiotics that are prescribe for both respiratory and non-respiratory infections as second generation cephalosporins were not included in our analysis, however, no increase in trend was observed for second generation cephalosporins throughout the study period. Quinolones and tetracyclines were not included since they are approved in the ambulatory setting for children > 16 years old and > 8 years old, respectively. Other antibiotics (erythromycin, clarithromycin, and clindamycin) constitutes only < 3% of dispensed antibiotics prescriptions in our community [20].

### Analysis

This is a population-based cohort study evaluating changes in clinic visits and dispensed medication rates during the COVID-19 pandemic (February 2020- January 2021) compared to four pre-COVID-19 pandemic years (February 2016- January 2020). Monthly incidence rates for respiratory and non-respiratory diagnoses and drugs (per 1,000 insured children) were calculated for each age group (children < 5, 5–17 and < 18 years) and ethnicity (Bedouin, Jewish and all children) (Online Resource 1.1–10). For the pre-pandemic period, we calculated mean monthly incidence rates with 95% CIs. Yearly and monthly rate ratios were calculated to compare between the two periods. Rate ratios were adjusted for age and ethnicity using the Mantel–Haenszel method. Relative reduction ratios were calculated to estimate the differences in reduction rates between respiratory and non-respiratory diagnoses and drugs. Data were analyzed using SPSS 26.0 and R 4.03. A *P* value of < 0.05 was considered statistically significant.

## Results

During the 5 study years, 5,588,702 clinic visits were recorded for children < 18 years: 2,845,857 (50.9%) for children < 5 years and 2,742,845 (49.1%) for children 5–17 years.

### COVID-19 number of national cases and non-pharmaceutical interventions (NPIs)

**Fig. 1:**
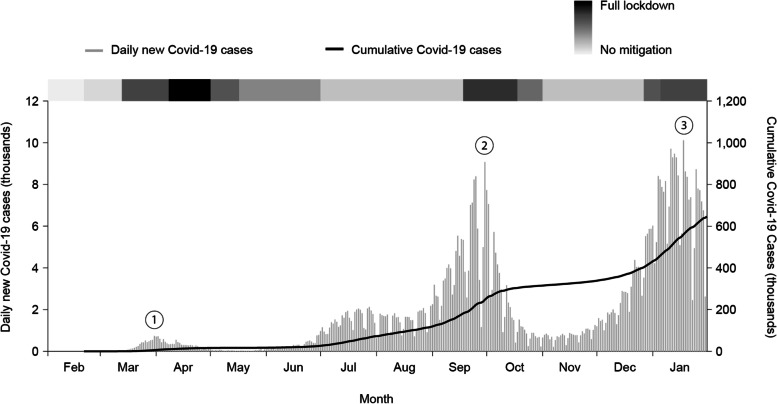
COVID-19 number of national cases and non-pharmaceutical interventions (NPIs)

The daily number of COVID-19 patients in Israel, nationwide, along with the major NPIs and COVID-19 immunization implementation are presented in Fig. 1.Three peaks in number of COVID-19 cases were noted during the study period, usually referred to as three waves. The first wave occurred in March 2020, and was followed by the first lockdown, which was the strictest NPI used. The second and the third peak, followed by the second and the third lockdowns, occurred in September and December 2020, respectively.

In late December 2020, the COVID-19 immunization program was implemented in Israel.

### Overall outpatient clinic visits

**Fig. 2:**
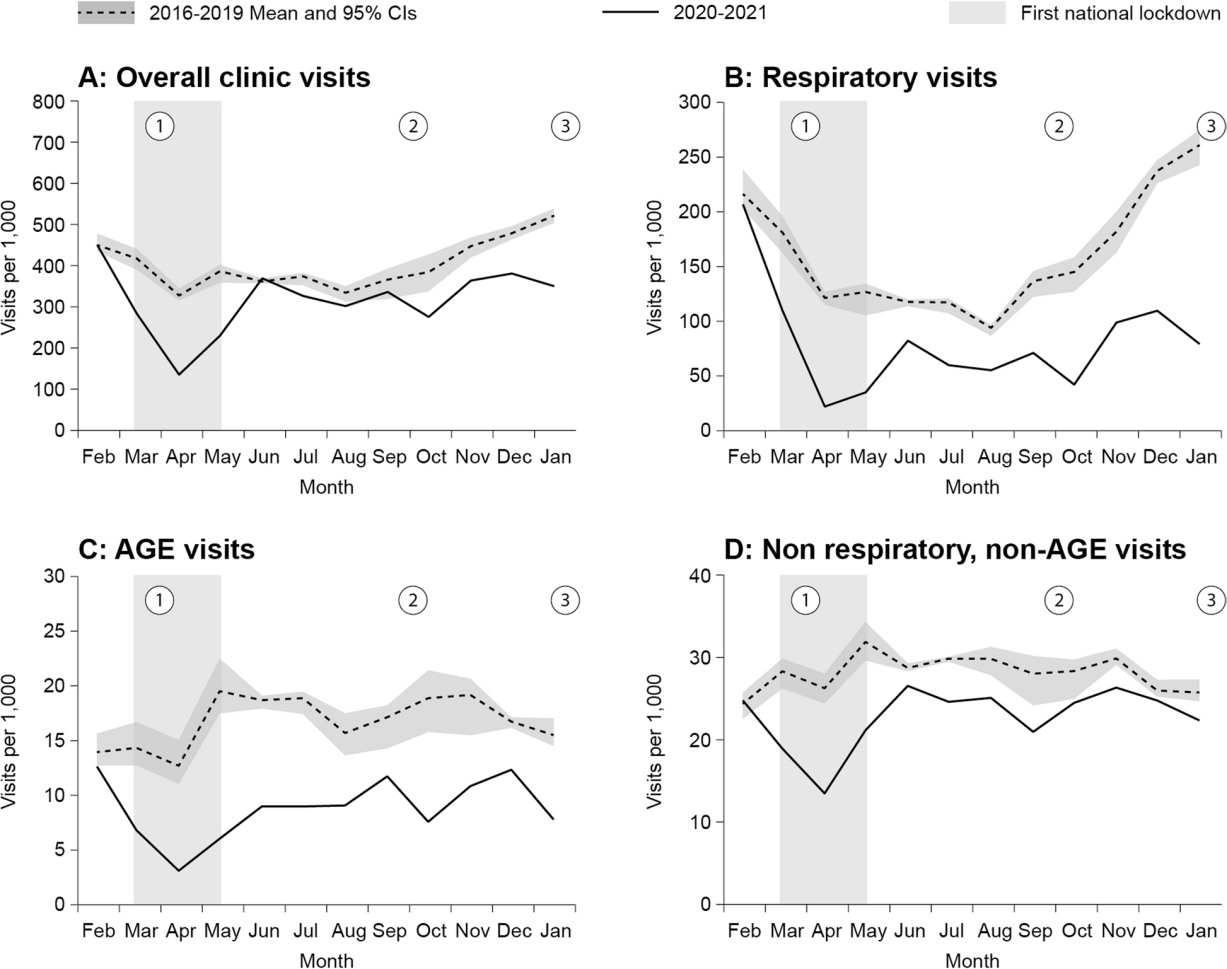
Monthly rates of overall, respiratory, acute gastroenteritis (AGE), and non-respiratory, non-AGE outpatient clinical visits in the pre-COVID-19 (2016–2019) and the COVID-19 period

The mean annual rate of all clinical visits in 2016–2019 (pre-COVID-19 period) declined by 21.6% (RR = 0.784; 95% CI: 0.783–0.786) in 2020–2021 (COVID-19 period) (Table 1, Fig. 2A). The decline was higher in Bedouin children (30.9%, RR = 0.69, 95% CI: 0.682–0.701) than Jewish children (16.3%, RR = 0.84, 95% CI: 0.830–0.845) and in children < 5 years (26.4%, RR = 0.74, 95% CI: 0.730–0.743) than children 5–17 years (16.9%, RR = 0.83, 95% CI: 0.822–0.845), (Online Resource 1.3, 1.4, 1.5, 1.8).

**Table 1:** Yearly rates, rate ratios and reductions 2020–2021 vs*.* 2016–2019 in children < 18 years old

	**Mean yearly rate**	**Yearly rate**	**Rate Ratio**	**Reduction**
	**Feb 2016-Jan 2020**	**Feb 2020-Jan 2021**		
**Visits**^a^				
Overall clinic visits	4852.1 ± 99.4	3804.1	0.784 (0.783 to 0.786)	21.6%
Respiratory visits	1938.3 ± 85.6	970.7	0.501 (0.499 to 0.503)	49.9%
URI	1459.4 ± 64.5	730.7	0.501 (0.499 to 0.504)	49.9%
LRI	158.3 ± 6.2	66.3	0.419 (0.413 to 0.426)	58.1%
AOM	281 ± 14.6	149.6	0.533 (0.528 to 0.539)	46.7%
Asthma	39.5 ± 2.2	24	0.608 (0.592 to 0.625)	39.2%
AGE visits	201.2 ± 11.3	106	0.527 (0.521 to 0.534)	47.3%
Non-respiratory, non-AGE visits	540.7 ± 19.9	381.5	0.706 (0.701 to 0.711)	29.4%
UTI	23.1 ± 0.9	18.9	0.818 (0.792 to 0.843)	18.2%
Epilepsy	15.8 ± 0.9	17.1	1.082 (1.046 to 1.119)	-8.2%
Trauma	242.1 ± 8.9	193.9	0.801 (0.794 to 0.809)	19.9%
SSTI	57.5 ± 1.4	45	0.782 (0.766 to 0.798)	21.8%
**Prescriptions**^a^				
All respiratory prescriptions	1577.1 ± 93.5	838.1	0.532 (0.53 to 0.535)	46.8%
Respiratory antibiotics prescriptions	766.4 ± 53.7	387.8	0.507 (0.504 to 0.51)	49.3%
Amoxicillin/Amoxicillin clavulanate	629 ± 44.3	316.6	0.504 (0.5 to 0.508)	49.6%
Azithromycin	126.1 ± 7.8	66.1	0.525 (0.517 to 0.533)	47.5%
Ceftriaxone	11.3 ± 1.6	5.1	0.455 (0.429 to 0.482)	54.5%
Respiratory non-antibiotic prescriptions	810.8 ± 40.6	450.3	0.556 (0.553 to 0.56)	44.4%
Asthma inhalators and solutions	270.7 ± 6.2	164.8	0.609 (0.603 to 0.615)	39.1%
Throat relief and nasal congestion	540.1 ± 37.1	285.5	0.53 (0.525 to 0.534)	47.0%
All non-respiratory prescriptions	118.6 ± 3.6	110.1	0.928 (0.916 to 0.94)	7.2%
Non-respiratory antibiotics prescriptions	56.1 ± 3	47.7	0.849 (0.832 to 0.866)	15.1%
OFGC	47.5 ± 3.2	41.7	0.876 (0.858 to 0.895)	12.4%
TMP/SMX	8.6 ± 0.8	6	0.697 (0.66 to 0.736)	30.3%
Non-respiratory, non-antibiotics prescriptions				
Anti-epileptic	62.5 ± 1	62.4	0.998 (0.981 to 1.016)	0.2%

*URI* upper respiratory tract infection, *LRI* lower respiratory tract infection, *AOM* acute otitis media, *AGE* acute gastroenteritis, *UTI* urinary tract infection, *SSTI* skin and soft tissue infection, *OFGC* oral first generation cephalosporins, *TMP/SMX* trimethoprim/sulfamethoxazole

^a^per 1,000 children

Reduction rates (comparing the pre-COVID-19 and the COVID-19 periods) were the highest in the months of April and May (59% and 40% reduction, respectively), following the first lockdown. In contrast, no declines were noted in February (prior to the first lockdown), and in June, following the relief of most NPI measures (Online Resource 1.1).

### Respiratory visits

The mean annual rate of all respiratory visits in the pre-COVID-19 period declined by 49.9% (RR = 0.501; 95% CI: 0.499–0.503) during the COVID-19 period, the declines were similar in both ethnic and age groups. (Fig. 2B, Online Resource 1.3, 1.4, 1.5, 1.8). The respective declines for the rates of URI, LRI, AOM and asthma were 49.9%, 58.1%, 46.7% and 39.2% (Online Resource 2.1).

Reduction rates (comparing the pre-COVID-19 and the COVID-19 periods) were the highest in the months of April and May (82% and 72% reductions, respectively), following the first lockdown (Online Resource 1.1).

### Acute gastroenteritis visits

The mean annual rate of AGE visits in the pre-COVID-19 period declined by 47.3% (RR = 0.527; 95% CI: 0.521–0.534) in the COVID-19 period, the declines were similar in both ethnic and age groups (Fig. 2C, Online Resource 1.3, 1.4, 1.5, 1.8). Reduction rates (comparing the pre-COVID-19 and the COVID-19 periods) were highest in the months of April and May (76% and 69% reductions, respectively), following the first lockdown (Online Resource 1.1).

### Non-respiratory, non-AGE visits

The mean annual rate of all four non-respiratory, non-AGE visits in the pre-COVID-19 period declined by 18.8% (RR = 0.812; 95% CI: 0.806–0.819) in the COVID-19 period Fig. 2D, the declines were higher in Bedouin children (26.4%, RR = 0.74; CI: 0.697–0.777) than Jewish children (13.2%, RR = 0.868; CI: 0.827–0.911) and in children 5–17 years old (22.9%, RR = 0.771; CI: 0.736–0.807) than children < 5 years (13%, RR = 0.87; CI: 0.821–0.922), Online Resource 1.3, 1.4, 1.5, 1.8. The respective declines for the rates of UTI, trauma, and SSTI were 18.2%, 19.9%, and 21.8%. An increase of 8.2% in the rate of epilepsy visits was observed (Table 1, Online Resource 2.2).

Reduction rates (comparing the pre-COVID-19 and the COVID-19 periods) were highest in the months of April and May (49% and 33% reductions, respectively), following the first lockdown (Online Resource 1.1).

### Dispensed medication prescription rates

#### All respiratory drugs

**Fig. 3:**
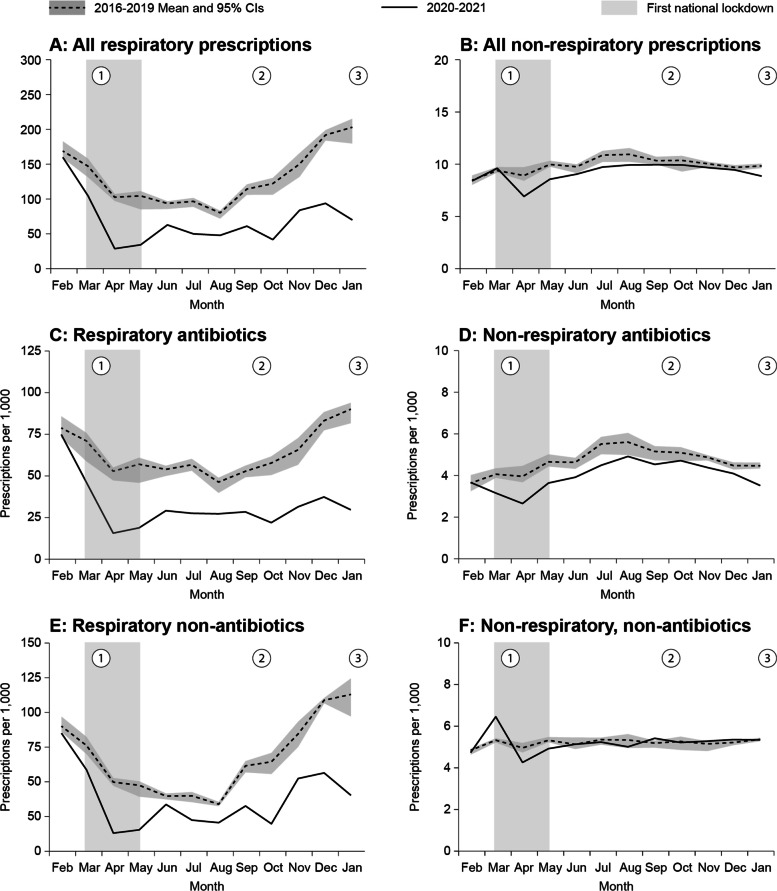
Monthly rates of respiratory, and non-respiratory dispensed drugs prescriptions in the pre-COVID-19 (2016–2019) and the COVID-19 period

The mean annual rate of all respiratory medication prescriptions in the pre-COVID-19 period declined by 46.8% (RR = 0.532; 95% CI: 0.530–0.535) in the COVID-19 period, the declines were similar in both ethnic and age groups. (Table 1, Fig. 3A, Online Resource 1.3, 1.4, 1.5, 1.8). Reduction rates (comparing the pre-COVID-19 and the COVID-19 periods) were highest in the months of April and May (72% and 67% reductions, respectively), following the first lockdown (Online Resource 1.1).

The mean annual rate of all respiratory antibiotics prescriptions in the pre-COVID-19 period declined by 49.3% (RR = 0.507; 95% CI: 0.504–0.510) in the COVID-19 period (Table 1, Fig. 3C). The respective declines for the rates of Amoxicillin / Amoxicillin clavulanate, Azithromycin and Ceftriaxone were 49.6%, 47.5% and 54.5%, respectively (Table 1, Online Resource 2.3).

The mean annual rate of all respiratory non-antibiotics medication prescriptions in the pre-COVID-19 period declined by 44.4% (RR = 0.556; 95% CI: 0.553–0.560) during the COVID-19 period (Table 1, Fig. 3E). The respective declines in the rates of use of asthma inhalators and solutions, and throat relief and nasal congestion were 39.1% and 47.0%. (Table 1, Online Resource 2.4).

#### Other, non-respiratory drugs

The mean annual rate of all non-respiratory medication prescriptions in the pre-COVID-19 period declined by 7.2% (RR = 0.928; 95% CI: 0.916–0.940) in the COVID-19 period (Table 1, Fig. 3B). Reduction rates (comparing the pre-COVID-19 and the COVID-19 periods) were highest in the months of April and May (23% and 14% reductions, respectively), following the first lockdown (Online Resource 1.1). The mean annual rate of all non-respiratory antibiotics prescriptions in the pre-COVID-19 period declined by 15.1% (RR = 0.849; 95% CI: 0.832–0.866) in the COVID-19 period (Table 1, Fig. 3D). The respective declines for the rates of oral first generation cephalosporins and trimethoprim/sulfamethoxazole were 12.4% and 30.3%, respectively (Table 1, Online Resource 2.5).

The mean annual rate of non-respiratory, non-antibiotics prescriptions (antiepileptic drugs) in the pre-COVID-19 period declined by 0.2% (RR = 0.998; 95% CI: 0.981–1.016) in the COVID-19 period. Moreover, in Jewish children < 5 years and in Bedouin children 5–17 years an increase in non-respiratory, non-antibiotics prescriptions was observed in the COVID-19 period. (Table 1, Fig. 3F, Online Resource 1.6, 1.10).

### Reduction ratios comparing respiratory and non-respiratory, non-AGE visits and drugs

The reduction ratio comparing all respiratory visits to non-respiratory, non-AGE visits was 0.62 (*P* < 0.001). The reduction ratio comparing all respiratory drugs to non-respiratory, non-AGE drugs was 0.57 (*P* < 0.001).

## Discussion

The COVID-19 pandemic resulted in a substantial reduction in overall outpatient clinic visits in children < 18 years in southern Israel compared to the four previous years. This reduction was mainly driven by the reduction in respiratory visits with only a mild reduction seen for non-respiratory visits. Furthermore, a greater reduction was seen for dispensed respiratory drugs prescription rates compared to non-respiratory drugs rates. These trends were similar in both ethnic and age groups.

Two processes unique to the COVID-19 pandemic might partially explain our findings: First, mitigation measures that were taken to control COVID-19 transmission, including closure of all educational facilities, movement and travel restrictions and discontinuation of nonessential work and commerce. These measures resulted in a change in human behavior, limited social encounters and reduced opportunities for person-to-person transmission. In addition, mandatory wearing of a mask and increasing awareness of hand hygiene minimized human exposures, especially with regard to infections transmitted via droplet and the fecal–oral route [23–25]. Thus, the greatest reductions in respiratory visits, AGE visits and in dispensed respiratory drugs prescription rates were seen during the months of April and May 2020, when a complete very strict nationwide lockdown was enforced by the Israeli MOH.

Second, the reductions in overall respiratory visits (50%) and in all respiratory drugs (47%) cannot be explained solely by mitigation measures, taking into account the large proportion of children under the age of 7 years in our cohort, who were not required to wear masks. In addition, half of the study population were Bedouin children living within large families with limited ability to fully maintain social distancing [19]. Finally, mitigation measures in Israel changed frequently throughout 2020, with intermittent periods of tightening and relieving restrictions, while the reductions in respiratory visits and respiratory drugs prescription rates were observed throughout the period with no correlation to mitigation stringency (except during the first lockdown) and have been sustained during the fall and early winter months and during the second and third COVID-19 waves. All of these factors allude to another factor impacting clinic visits rate, which is the profound disruption in the seasonal activity of respiratory viruses that accompanied the COVID-19 pandemic worldwide. The disappearance of seasonal respiratory viruses, mainly RSV and influenza, was first reported from countries in the southern hemisphere and later from North America, Europe, and Israel [8, 26]. Apart from rhinovirus and adenovirus, which are common in Israel throughout the year, RSV, influenza viruses, and human metapneumovirus (hMPV) have a fall to early-spring seasonality and parainfluenza virus has a summer to fall seasonality. However, since April 2020, RSV, influenza viruses, hMPV and parainfluenza had a complete suppression that continued through December 2020 for parainfluenza and February 2021 for RSV, influenza viruses and hMPV. In contrast, adenovirus and rhinovirus activity was as expected (except during the first lockdown period, March–April 2020) [27]. Co-circulation of respiratory viruses might be affected both by host susceptibility and by virus-virus interactions, while competing on shared biological niches. A mechanism of viral interference was already suggested during influenza virus A (H1N1) pandemic in 2009 (pdm09). However, data comparing same year-periods did not support this hypothesis and respiratory viruses other than H1N1pdm09 showed epidemiological patterns similar to previous years [28].

The lack of seasonal respiratory virus activity during the COVID-19 pandemic might also affect noninfectious respiratory diagnoses, such as asthma, since respiratory viruses are frequently the trigger of asthma exacerbations in young children [29].

Antibiotics for respiratory infections are the most commonly prescribed medications in children [30, 31] and overuse of antibiotics resulting in increased antibiotic resistance is a major global concern [32]. A reduction in antibiotic use was observed by previous studies conducted during the first months of COVID-19 pandemic, mainly for antibiotics that are used for the treatment of respiratory infections [14, 15]. A major concern during COVID-19 pandemic was that increase in telemedicine might result in antibiotics overuse [33–35]. In our study, the reductions noted in dispensed respiratory drugs prescription rates mirrored that of the respiratory visits. A further study comparing face-to-face vs. remote visits drugs prescription rates is required.

Our study is the first population-based study that combined data on both outpatient clinic visits, visits diagnoses and dispensed antibiotic and non-antibiotic prescription rates that was conducted throughout 12 months of the COVID-19 pandemic, including baseline data starting four years prior to the pandemic. In addition, we included numerous control outcomes for both visits diagnoses and drugs to overcome bias. Visits due to convulsions, which are non-respiratory non-infectious, increased comparing COVID-19 to pre-COVID-19 period and antiepileptic drugs remained essentially unchanged, thus adding strength to our findings.

Limitations of the current study include methodology that relies on coding reports, and, therefore, results might be influenced by coding variations between individual providers or clinics, and inherently may contain missing or inaccurate data. Moreover, the shift towards telemedicine that occurred during 2020 may underestimate diagnoses that warrant ear, nose, and throat examination. Additionally, we cannot rule out other factors which could potentially play a role in diagnosis and medication prescription, although no significant changes in guidelines occurred, and no limitations for the use of specific medications were imposed during the study period.

## Conclusions

In conclusion, respiratory outpatient visits and dispensed respiratory drugs prescription rates significantly decreased in children < 18 years in southern Israel during the COVID-19 pandemic period compared to the four previous years. The reductions in non-respiratory visits and non-respiratory drug rates were much smaller. Same trends were seen in all ethnic and age groups.

Importantly, the reduction in outpatient clinic visits may be temporal, since a decrease in COVID-19 cases might result in resurgence of seasonal respiratory viruses, as was seen with RSV in Australian children [34]. Therefore, ongoing surveillance is needed to evaluate the long-term effect of COVID-19 pandemic on pediatric outpatient healthcare utilization and antibiotic use.

## Supplementary Information

**Additional file 1.**

**Additional file 2.**

## Data Availability

The datasets generated and/or analyzed during the current study are not publicly available due to patient confidentiality.

## Abbreviations

AGE: Acute gastroenteritis
AOM: Acute otitis media
COVID-19: Coronavirus disease 2019
HMO: Health maintenance organization
hMPV: Human metapneumovirus
LRI: Lower respiratory tract infection
MOH: Ministry of Health
NPI: Non-pharmaceutical intervention
RSV: Respiratory syncytial virus
SSTI: Skin and soft tissue infection
URI: Upper respiratory tract infection
UTI: Urinary tract infection
