# Supplementary material for: Disproportionate reduction in respiratory vs. non-respiratory outpatient clinic visits and antibiotic use in children during the COVID-19 pandemic
Source: BMC Pediatr. 2022 May 6;22:254. doi: 10.1186/s12887-022-03315-0 (PMC9073498; doi:10.1186/s12887-022-03315-0)
Supplement: Supplementary file 1 — Additional file 1. [file 12887_2022_3315_MOESM1_ESM.docx]

**ONLINE RESOURCE 1: SUPPLEMENTARY TABLES**

**Disproportionate Reduction in Respiratory Vs. Non-Respiratory Outpatient Clinic Visits and Antibiotic Use in Children During the COVID-19 Pandemic: Implications for the Roles of Human Behavior and SARS-Cov-2 Circulation Effect**

**Noga Givon-Lavi*,PhD^1, 2^ Dana Danino*, MD^1, 2^ Bart Adriaan Van Der Beek,^1^ Amir Sharf,^3^ David Greenberg, MD^1,2^ Shalom Ben-Shimol, MD^1, 2^**

^1^ Faculty of Health Sciences, Ben-Gurion University of the Negev, Beer-Sheva, Israel

^2^ The Pediatric Infectious Disease Unit, Soroka University Medical Center, Beer-Sheva, Israel

^3^Economics and Data Analysis Department, Clalit HMO South district, Beer-Sheva, Israel

*Both authors contributed equally to this manuscript

**Corresponding author:**

Dana Danino, MD

The Pediatric Infectious Disease Unit

Soroka University Medical Center

Beer-Sheva, Israel.

E-mail: danadanino@hotmail.com

Tel: 972-8-6400547

Fax: 972-8-6232334

|  | **Feb** | **Mar** | **Apr** | **May** | **Jun** | **Jul** | **Aug** | **Sep** | **Oct** | **Nov** | **Dec** | **Jan** |
| --- | --- | --- | --- | --- | --- | --- | --- | --- | --- | --- | --- | --- |
| Overall clinic visits | 1.00 (1.00 to 1.01) | 0.68 (0.67 to 0.69) | 0.41 (0.41 to 0.42) | 0.60 (0.59 to 0.60) | 1.02 (1.01 to 1.03) | 0.87 (0.87 to 0.88) | 0.90 (0.90 to 0.91) | 0.92 (0.91 to 0.93) | 0.72 (0.71 to 0.72) | 0.81 (0.81 to 0.82) | 0.79 (0.79 to 0.80) | 0.67 (0.67 to 0.68) |
| Respiratory visits | 0.96 (0.95 to 0.97) | 0.61 (0.60 to 0.61) | 0.18 (0.18 to 0.19) | 0.28 (0.27 to 0.28) | 0.70 (0.69 to 0.71) | 0.51 (0.50 to 0.52) | 0.59 (0.58 to 0.60) | 0.52 (0.51 to 0.53) | 0.29 (0.28 to 0.30) | 0.54 (0.54 to 0.55) | 0.46 (0.46 to 0.47) | 0.30 (0.30 to 0.31) |
| URI | 0.97 (0.96 to 0.98) | 0.61 (0.60 to 0.62) | 0.17 (0.17 to 0.18) | 0.27 (0.26 to 0.27) | 0.72 (0.71 to 0.73) | 0.49 (0.48 to 0.50) | 0.58 (0.57 to 0.59) | 0.52 (0.51 to 0.53) | 0.27 (0.27 to 0.28) | 0.56 (0.55 to 0.57) | 0.47 (0.46 to 0.48) | 0.30 (0.29 to 0.30) |
| LRI | 0.85 (0.82 to 0.88) | 0.63 (0.61 to 0.66) | 0.14 (0.12 to 0.15) | 0.23 (0.21 to 0.25) | 0.64 (0.60 to 0.68) | 0.51 (0.48 to 0.55) | 0.50 (0.46 to 0.54) | 0.42 (0.40 to 0.45) | 0.21 (0.19 to 0.22) | 0.39 (0.37 to 0.41) | 0.33 (0.31 to 0.34) | 0.24 (0.23 to 0.26) |
| AOM | 0.94 (0.92 to 0.96) | 0.58 (0.56 to 0.60) | 0.22 (0.21 to 0.24) | 0.31 (0.29 to 0.32) | 0.61 (0.59 to 0.64) | 0.59 (0.57 to 0.62) | 0.66 (0.64 to 0.69) | 0.58 (0.56 to 0.60) | 0.41 (0.39 to 0.43) | 0.54 (0.52 to 0.56) | 0.52 (0.50 to 0.54) | 0.36 (0.34 to 0.37) |
| Asthma | 1.00 (0.93 to 1.08) | 0.69 (0.64 to 0.75) | 0.35 (0.31 to 0.40) | 0.54 (0.48 to 0.60) | 0.87 (0.79 to 0.95) | 0.63 (0.57 to 0.71) | 0.58 (0.52 to 0.65) | 0.58 (0.52 to 0.64) | 0.38 (0.34 to 0.43) | 0.69 (0.64 to 0.75) | 0.53 (0.48 to 0.57) | 0.48 (0.44 to 0.53) |
| AGE visits | 0.91 (0.87 to 0.94) | 0.48 (0.45 to 0.50) | 0.24 (0.23 to 0.26) | 0.31 (0.29 to 0.33) | 0.48 (0.46 to 0.50) | 0.48 (0.46 to 0.50) | 0.58 (0.55 to 0.61) | 0.69 (0.66 to 0.71) | 0.40 (0.38 to 0.42) | 0.57 (0.54 to 0.59) | 0.74 (0.71 to 0.77) | 0.50 (0.48 to 0.53) |
| Non-respiratory, non-AGE visits | 1.02 (0.99 to 1.05) | 0.67 (0.65 to 0.69) | 0.51 (0.50 to 0.53) | 0.67 (0.65 to 0.68) | 0.92 (0.90 to 0.95) | 0.82 (0.80 to 0.85) | 0.84 (0.82 to 0.87) | 0.75 (0.73 to 0.77) | 0.86 (0.84 to 0.89) | 0.88 (0.86 to 0.91) | 0.95 (0.93 to 0.98) | 0.87 (0.84 to 0.89) |
| UTI | 0.96 (0.87 to 1.06) | 0.67 (0.60 to 0.75) | 0.62 (0.55 to 0.71) | 0.75 (0.67 to 0.84) | 0.96 (0.86 to 1.07) | 0.77 (0.69 to 0.85) | 0.76 (0.68 to 0.85) | 0.83 (0.74 to 0.93) | 0.88 (0.79 to 0.98) | 1.04 (0.95 to 1.15) | 0.88 (0.80 to 0.98) | 0.70 (0.62 to 0.78) |
| Epilepsy | 1.20 (1.08 to 1.34) | 0.91 (0.80 to 1.03) | 0.66 (0.56 to 0.78) | 0.91 (0.80 to 1.04) | 1.38 (1.23 to 1.55) | 0.91 (0.80 to 1.02) | 1.05 (0.93 to 1.18) | 1.17 (1.04 to 1.31) | 1.35 (1.21 to 1.50) | 1.44 (1.30 to 1.60) | 1.13 (1.01 to 1.27) | 0.89 (0.80 to 0.99) |
| Trauma | 1.01 (0.97 to 1.04) | 0.63 (0.61 to 0.65) | 0.48 (0.46 to 0.50) | 0.64 (0.62 to 0.66) | 0.92 (0.89 to 0.95) | 0.86 (0.83 to 0.88) | 0.85 (0.83 to 0.88) | 0.73 (0.70 to 0.75) | 0.84 (0.81 to 0.87) | 0.84 (0.82 to 0.87) | 0.99 (0.96 to 1.03) | 0.90 (0.87 to 0.93) |
| SSTI | 1.04 (0.97 to 1.12) | 0.79 (0.74 to 0.85) | 0.60 (0.55 to 0.65) | 0.70 (0.65 to 0.75) | 0.81 (0.75 to 0.87) | 0.71 (0.66 to 0.76) | 0.78 (0.73 to 0.83) | 0.72 (0.67 to 0.77) | 0.84 (0.78 to 0.89) | 0.84 (0.79 to 0.90) | 0.78 (0.72 to 0.83) | 0.83 (0.77 to 0.89) |
| All respiratory prescription | 0.95 (0.94 to 0.96) | 0.71 (0.70 to 0.72) | 0.28 (0.27 to 0.29) | 0.33 (0.32 to 0.34) | 0.67 (0.66 to 0.68) | 0.52 (0.51 to 0.53) | 0.60 (0.59 to 0.61) | 0.53 (0.53 to 0.54) | 0.34 (0.34 to 0.35) | 0.56 (0.55 to 0.57) | 0.49 (0.48 to 0.50) | 0.35 (0.34 to 0.35) |
| Respiratory antibiotics prescriptions | 0.95 (0.94 to 0.97) | 0.65 (0.64 to 0.66) | 0.30 (0.29 to 0.31) | 0.33 (0.32 to 0.34) | 0.54 (0.53 to 0.55) | 0.49 (0.47 to 0.50) | 0.59 (0.58 to 0.61) | 0.54 (0.53 to 0.55) | 0.38 (0.37 to 0.39) | 0.48 (0.47 to 0.49) | 0.45 (0.44 to 0.46) | 0.33 (0.32 to 0.34) |
| Amoxicillin/  Amoxicillin clavulanate | 0.94 (0.93 to 0.96) | 0.63 (0.62 to 0.64) | 0.29 (0.28 to 0.30) | 0.33 (0.32 to 0.34) | 0.54 (0.53 to 0.56) | 0.50 (0.48 to 0.51) | 0.61 (0.59 to 0.63) | 0.54 (0.52 to 0.55) | 0.39 (0.38 to 0.40) | 0.47 (0.46 to 0.48) | 0.44 (0.43 to 0.45) | 0.32 (0.31 to 0.33) |
| Azithromycin | 1.02 (0.98 to 1.06) | 0.77 (0.74 to 0.81) | 0.31 (0.29 to 0.34) | 0.34 (0.31 to 0.36) | 0.54 (0.51 to 0.58) | 0.45 (0.42 to 0.48) | 0.51 (0.48 to 0.54) | 0.54 (0.51 to 0.58) | 0.34 (0.32 to 0.37) | 0.53 (0.50 to 0.56) | 0.51 (0.48 to 0.53) | 0.36 (0.34 to 0.38) |
| Ceftriaxone | 0.85 (0.74 to 0.98) | 0.54 (0.45 to 0.64) | 0.25 (0.19 to 0.33) | 0.28 (0.22 to 0.36) | 0.35 (0.27 to 0.44) | 0.38 (0.31 to 0.47) | 0.53 (0.43 to 0.64) | 0.46 (0.37 to 0.57) | 0.34 (0.27 to 0.43) | 0.47 (0.38 to 0.57) | 0.50 (0.42 to 0.60) | 0.40 (0.33 to 0.48) |
| Asthma inhalators and solutions | 1.01 (0.98 to 1.04) | 0.97 (0.95 to 1.00) | 0.36 (0.34 to 0.38) | 0.39 (0.37 to 0.41) | 0.79 (0.75 to 0.82) | 0.65 (0.62 to 0.68) | 0.58 (0.56 to 0.61) | 0.61 (0.59 to 0.64) | 0.34 (0.32 to 0.35) | 0.63 (0.61 to 0.65) | 0.53 (0.51 to 0.54) | 0.45 (0.43 to 0.46) |
| Throat relief and nasal congestion | 0.92 (0.90 to 0.94) | 0.67 (0.65 to 0.68) | 0.21 (0.20 to 0.22) | 0.29 (0.28 to 0.31) | 0.88 (0.86 to 0.91) | 0.51 (0.49 to 0.53) | 0.62 (0.60 to 0.64) | 0.49 (0.48 to 0.51) | 0.29 (0.28 to 0.30) | 0.62 (0.60 to 0.63) | 0.51 (0.5 to 0.53) | 0.32 (0.31 to 0.32) |
| All non-respiratory prescriptions | 0.99 (0.94 to 1.04) | 1.02 (0.98 to 1.07) | 0.77 (0.74 to 0.82) | 0.86 (0.82 to 0.90) | 0.93 (0.89 to 0.97) | 0.89 (0.86 to 0.93) | 0.91 (0.87 to 0.95) | 0.96 (0.92 to 1.00) | 0.96 (0.92 to 1.00) | 0.96 (0.92 to 1.01) | 0.97 (0.93 to 1.02) | 0.90 (0.86 to 0.94) |
| Non-respiratory antibiotics prescriptions | 1.01 (0.94 to 1.08) | 0.78 (0.72 to 0.84) | 0.67 (0.62 to 0.73) | 0.78 (0.73 to 0.84) | 0.84 (0.79 to 0.90) | 0.81 (0.76 to 0.87) | 0.88 (0.82 to 0.93) | 0.88 (0.82 to 0.94) | 0.92 (0.87 to 0.98) | 0.90 (0.84 to 0.96) | 0.91 (0.85 to 0.98) | 0.79 (0.73 to 0.85) |
| OFGC | 1.08 (1.00 to 1.17) | 0.80 (0.74 to 0.87) | 0.69 (0.64 to 0.76) | 0.84 (0.78 to 0.91) | 0.89 (0.83 to 0.96) | 0.83 (0.77 to 0.89) | 0.90 (0.84 to 0.96) | 0.91 (0.85 to 0.97) | 0.95 (0.89 to 1.02) | 0.92 (0.86 to 0.98) | 0.91 (0.85 to 0.98) | 0.79 (0.73 to 0.85) |
| TMP/SMX | 0.69 (0.56 to 0.84) | 0.67 (0.56 to 0.81) | 0.55 (0.44 to 0.68) | 0.48 (0.38 to 0.59) | 0.61 (0.50 to 0.74) | 0.71 (0.59 to 0.86) | 0.74 (0.61 to 0.89) | 0.69 (0.57 to 0.84) | 0.74 (0.62 to 0.89) | 0.78 (0.65 to 0.94) | 0.92 (0.78 to 1.09) | 0.79 (0.66 to 0.94) |
| Non-respiratory, non-antibiotics prescriptions | 0.98 (0.92 to 1.04) | 1.21 (1.14 to 1.28) | 0.86 (0.80 to 0.92) | 0.92 (0.87 to 0.98) | 1.00 (0.94 to 1.06) | 0.98 (0.92 to 1.04) | 0.94 (0.88 to 1.00) | 1.04 (0.98 to 1.11) | 0.99 (0.93 to 1.05) | 1.03 (0.97 to 1.09) | 1.03 (0.97 to 1.09) | 1.00 (0.94 to 1.06) |
|  |  |  |  |  |  |  |  |  |  |  |  |  |
| URI, upper respiratory tract infection; LRI, lower respiratory tract infection; AOM, acute otitis media; AGE, acute gastroenteritis; SSTI, skin and soft tissue infection; OFGC, oral first generation cephalosporins; TMP/SMX, trimethoprim/sulfamethoxazole | | | | | | | | | | | | |
|  | | | | | | | | | | | | |
|  | | | | | | | | | | | | |

**Online Resource 1.1: Monthly rate ratios 2020-2021 vs. 2016-2019 in children <18 years old**

**Online Resource 1.2: Yearly rates and rate ratios 2020-2021 vs*.* 2016-2019, adjusted for age and ethnicity in children <18 years old**

|  |  | **Mean yearly rate**  **Feb 2016-Jan 2020** | **Yearly rate**  **Feb 2020-Jan 2021** | **Rate Ratio*** | **Reduction*** |
| --- | --- | --- | --- | --- | --- |
|  |  |  |  |  |  |
|  |  |  | **Visits** |  |  |
|  |  |  |  |  |  |
| Overall clinic visits^˧^ |  | 4852.1±99.4 | 3804.1 | 0.783 (0.777 to 0.789) | 21.7% |
| Respiratory visits^˧^ |  | 1938.3±85.6 | 970.7 | 0.504 (0.496 to 0.512) | 49.6% |
|  | URI | 1459.4±64.5 | 730.7 | 0.504 (0.494 to 0.514) | 49.6% |
|  | LRI | 158.3±6.2 | 66.3 | 0.429 (0.402 to 0.457) | 57.1% |
|  | AOM | 281±14.6 | 149.6 | 0.534 (0.51 to 0.558) | 46.6% |
|  | Asthma | 39.5±2.2 | 24 | 0.614 (0.546 to 0.691) | 38.6% |
| AGE visits^˧^ |  | 201.2±11.3 | 106 | 0.529 (0.501 to 0.558) | 47.1% |
| Non-respiratory, non-AGE visits^˧^ |  | 540.7±19.9 | 381.5 | 0.807 (0.778 to 0.837) | 19.3% |
|  | UTI | 23.1±0.9 | 18.9 | 0.815 (0.708 to 0.938) | 18.5% |
|  | Epilepsy | 15.8±0.9 | 17.1 | 1.069 (0.912 to 1.253) | -6.9% |
|  | Trauma | 242.1±8.9 | 193.9 | 0.799 (0.765 to 0.834) | 20.1% |
|  | SSTI | 57.5±1.4 | 45 | 0.768 (0.702 to 0.841) | 23.2% |
|  |  |  |  |  |  |
|  |  |  | Prescriptions |  |  |
|  |  |  |  |  |  |
| All respiratory prescriptions^˧^ |  | 1577.1±93.5 | 838.1 | 0.539 (0.53 to 0.549) | 46.1% |
|  |  |  |  |  |  |
| Respiratory antibiotics prescriptions^˧^ |  | 766.4±53.7 | 387.8 | 0.515 (0.501 to 0.529) | 48.5% |
|  | Amoxicillin/  Amoxicillin clavulanate | 629±44.3 | 316.6 | 0.511 (0.496 to 0.527) | 48.9% |
|  | Azithromycin | 126.1±7.8 | 66.1 | 0.535 (0.5 to 0.573) | 46.5% |
|  | Ceftriaxone | 11.3±1.6 | 5.1 | 0.461 (0.358 to 0.594) | 53.9% |
| Respiratory non-antibiotic prescriptions^˧^ |  | 810.8±40.6 | 450.3 | 0.563 (0.549 to 0.578) | 43.7% |
|  | Asthma inhalators and solutions | 270.7±6.2 | 164.8 | 0.619 (0.593 to 0.647) | 38.1% |
|  | Throat relief and nasal congestion | 540.1±37.1 | 285.5 | 0.535 (0.517 to 0.552) | 46.5% |
| All non-respiratory prescriptions^˧^ |  | 118.6±3.6 | 110.1 | 0.919 (0.865 to 0.975) | 8.1% |
|  |  |  |  |  |  |
| Non-respiratory antibiotics prescriptions^˧^ |  | 56.1±3 | 47.7 | 0.838 (0.767 to 0.916) | 16.2% |
|  | OFGC | 47.5±3.2 | 41.7 | 0.865 (0.786 to 0.952) | 13.5% |
|  | TMP/SMX | 8.6±0.8 | 6 | 0.692 (0.545 to 0.878) | 30.8% |
| Non-respiratory, non-antibiotics prescriptions^˧^ | Anti-epileptic | 62.5±1 | 62.4 | 0.993 (0.915 to 1.078) | 0.7% |

*adjusted for age

^˧^per 1,000 children

URI, upper respiratory tract infection; LRI, lower respiratory tract infection; AOM, acute otitis media; AGE, acute gastroenteritis; UTI, urinary tract infection; SSTI, skin and soft tissue infection; OFGC, oral first generation cephalosporins; TMP/SMX, trimethoprim/sulfamethoxazole

**Online Resource 1.3: Yearly rates and rate ratios 2020-2021 vs. 2016-2019 in Jewish children < 18 years old, adjusted for age**

|  |  | Mean yearly rate  Feb 2016-Jan 2020 | Yearly rate  Feb 2020-Jan 2021 | Rate Ratio* | Reduction* |
| --- | --- | --- | --- | --- | --- |
|  |  |  |  |  |  |
|  |  |  | Visits |  |  |
|  |  |  |  |  |  |
| Overall clinic visits^˧^ |  | 5852.7±94.5 | 4858.5 | 0.837 (0.83 to 0.845) | 16.3% |
| Respiratory visits^˧^ |  | 2187.2±88.8 | 1106.1 | 0.514 (0.504 to 0.525) | 48.6% |
|  | URI | 1662.0±65.1 | 853.6 | 0.521 (0.509 to 0.534) | 47.9% |
|  | LRI | 172.1±6.6 | 65.0 | 0.388 (0.354 to 0.424) | 61.2% |
|  | AOM | 306.5±15.9 | 158.4 | 0.53 (0.499 to 0.563) | 47.0% |
|  | Asthma | 46.6±3.8 | 29.2 | 0.635 (0.547 to 0.736) | 36.5% |
| AGE visits^˧^ |  | 216.0±21.5 | 110.7 | 0.524 (0.488 to 0.564) | 47.6% |
| Non-respiratory, non-AGE visits^˧^ |  | 361.1±13.7 | 312.0 | 0.868 (0.827 to 0.911) | 13.2% |
|  | UTI | 26.8±1.2 | 22.7 | 0.857 (0.716 to 1.026) | 14.3% |
|  | Epilepsy | 16.4±0.8 | 19.3 | 1.175 (0.95 to 1.453) | -17.5% |
|  | Trauma | 262.2±11.6 | 223.5 | 0.855 (0.808 to 0.905) | 14.5% |
|  | SSTI | 55.7±2.0 | 46.4 | 0.84 (0.742 to 0.952) | 16.0% |
|  |  |  |  |  |  |
|  |  |  | Prescriptions |  |  |
|  |  |  |  |  |  |
| All respiratory prescriptions^˧^ |  | 1768.7±74.5 | 938.0 | 0.538 (0.525 to 0.551) | 46.2% |
|  |  |  |  |  |  |
| Respiratory antibiotics prescriptions^˧^ |  | 784.8±35.1 | 385.7 | 0.498 (0.479 to 0.517) | 50.2% |
|  | Amoxicillin/  Amoxicillin clavulanate | 637.9±26.4 | 316.3 | 0.501 (0.481 to 0.523) | 49.9% |
|  | Azithromycin | 139.1±8.4 | 66.5 | 0.487 (0.444 to 0.535) | 51.3% |
|  | Ceftriaxone | 7.8±0.7 | 2.8 | 0.375 (0.243 to 0.58) | 62.5% |
| Respiratory non-antibiotic prescriptions^˧^ |  | 983.9±40.0 | 552.3 | 0.57 (0.552 to 0.589) | 43.0% |
|  | Asthma inhalators and solutions | 378.3±7.1 | 226.5 | 0.611 (0.58 to 0.643) | 38.9% |
|  | Throat relief and nasal congestion | 605.6±40.8 | 325.8 | 0.545 (0.522 to 0.568) | 45.5% |
| All non-respiratory prescriptions^˧^ |  | 121.4±1.8 | 115.1 | 0.947 (0.873 to 1.028) | 5.3% |
|  |  |  |  |  |  |
| Non-respiratory antibiotics prescriptions^˧^ |  | 59.9±1.5 | 54.8 | 0.923 (0.821 to 1.038) | 7.7% |
|  | OFGC | 52.3±2.0 | 49.0 | 0.945 (0.833 to 1.071) | 5.5% |
|  | TMP/SMX | 7.6±0.6 | 5.8 | 0.774 (0.546 to 1.096) | 22.6% |
| Non-respiratory, non-antibiotics prescriptions^˧^ | Anti-epileptic | 61.5±2.0 | 60.3 | 0.971 (0.866 to 1.088) | 2.9% |

*adjusted for age

^˧^per 1,000 children

URI, upper respiratory tract infection; LRI, lower respiratory tract infection; AOM, acute otitis media; AGE, acute gastroenteritis; UTI, urinary tract infection; SSTI, skin and soft tissue infection; OFGC, oral first generation cephalosporins; TMP/SMX, trimethoprim/sulfamethoxazole

**Online Resource 1.4: Yearly rates and rate ratios 2020-2021 vs. 2016-2019 in Bedouin children <18 years old, adjusted for age**

|  |  | Mean yearly rate  Feb 2016-Jan 2020 | Yearly rate  Feb 2020-Jan 2021 | Rate Ratio* | Reduction* |
| --- | --- | --- | --- | --- | --- |
|  |  |  |  |  |  |
|  |  |  | Visits |  |  |
|  |  |  |  |  |  |
| Overall clinic visits^˧^ |  | 3726.4±129.1 | 2595.2 | 0.691 (0.682 to 0.701) | 30.9% |
| Respiratory visits^˧^ |  | 1608.8±95.0 | 797.2 | 0.489 (0.477 to 0.502) | 51.1% |
|  | URI | 1173.9±71.3 | 566.1 | 0.477 (0.462 to 0.492) | 52.3% |
|  | LRI | 143.7±7.9 | 70.4 | 0.48 (0.437 to 0.528) | 52.0% |
|  | AOM | 260.2±17.6 | 142.7 | 0.539 (0.504 to 0.576) | 46.1% |
|  | Asthma | 46.6±3.8 | 29.2 | 0.58 (0.478 to 0.704) | 42.0% |
| AGE visits^˧^ |  | 187.0±11.0 | 101.9 | 0.535 (0.494 to 0.579) | 46.5% |
| Non-respiratory, non-AGE visits^˧^ |  | 331.6±12.4 | 244.3 | 0.736 (0.697 to 0.777) | 26.4% |
|  | UTI | 19.4±1.4 | 14.6 | 0.752 (0.599 to 0.943) | 24.8% |
|  | Epilepsy | 15.3±0.7 | 14.5 | 0.946 (0.744 to 1.203) | 5.4% |
|  | Trauma | 235.0±10.0 | 171.7 | 0.731 (0.684 to 0.78) | 26.9% |
|  | SSTI | 61.9±2.3 | 43.5 | 0.699 (0.614 to 0.795) | 30.1% |
|  |  |  |  |  |  |
|  |  |  | Prescriptions |  |  |
|  |  |  |  |  |  |
| All respiratory prescriptions^˧^ |  | 1331.1±110.9 | 727.4 | 0.542 (0.526 to 0.557) | 45.8% |
|  |  |  |  |  |  |
| Respiratory antibiotics prescriptions^˧^ |  | 732.7±71.0 | 395 | 0.534 (0.513 to 0.556) | 46.6% |
|  | Amoxicillin/  Amoxicillin clavulanate | 607.3±60.5 | 319.8 | 0.522 (0.5 to 0.546) | 47.8% |
|  | Azithromycin | 112.6±9.1 | 68.5 | 0.598 (0.541 to 0.661) | 40.2% |
|  | Ceftriaxone | 12.8±2.4 | 6.7 | 0.516 (0.378 to 0.705) | 48.4% |
| Respiratory non-antibiotic prescriptions^˧^ |  | 598.4±40.3 | 332.4 | 0.551 (0.527 to 0.575) | 44.9% |
|  | Asthma inhalators and solutions | 149.3±8.5 | 97.2 | 0.643 (0.591 to 0.7) | 35.7% |
|  | Throat relief and nasal congestion | 449.1±32.2 | 235.2 | 0.52 (0.494 to 0.548) | 48.0% |
| All non-respiratory prescriptions^˧^ |  | 116.6±5.7 | 103.4 | 0.886 (0.811 to 0.968) | 11.4% |
|  |  |  |  |  |  |
| Non-respiratory antibiotics prescriptions^˧^ |  | 54.4±5.0 | 40.5 | 0.738 (0.645 to 0.845) | 26.2% |
|  | OFGC | 44.2±4.6 | 34.1 | 0.764 (0.658 to 0.887) | 23.6% |
|  | TMP/SMX | 10.2±1.4 | 6.5 | 0.627 (0.451 to 0.871) | 37.3% |
| Non-respiratory, non-antibiotics prescriptions^˧^ | Anti-epileptic | 62.2±0.8 | 62.8 | 1.017 (0.904 to 1.143) | -1.7% |
| *adjusted for ethnicity  ^˧^per 1,000 children  URI, upper respiratory tract infection; LRI, lower respiratory tract infection; AOM, acute otitis media; AGE, acute gastroenteritis; UTI, urinary tract infection; SSTI, skin and soft tissue infection; OFGC, oral first generation cephalosporins; TMP/SMX, trimethoprim/sulfamethoxazole | | | | | |
|  |  |  |  |  |  |

**Online Resource 1.5: Yearly rates and rate ratios 2020-2021 vs. 2016-2019, adjusted for ethnicity in children < 5 years old**

|  |  | Mean yearly rate  Feb 2016-Jan 2020 | Yearly rate  Feb 2020-Jan 2021 | Rate Ratio* | Reduction* |
| --- | --- | --- | --- | --- | --- |
|  |  |  |  |  |  |
|  |  |  | Visits |  |  |
|  |  |  |  |  |  |
| Overall clinic visits^˧^ |  | 7573.1±209.9 | 5543.1 | 0.736 (0.73 to 0.743) | 26.4% |
| Respiratory visits^˧^ |  | 3853.2±189.1 | 1893.6 | 0.496 (0.487 to 0.506) | 50.4% |
|  | URI | 2698±137.1 | 1342.1 | 0.502 (0.49 to 0.515) | 49.8% |
|  | LRI | 409.1±15.1 | 165.2 | 0.414 (0.385 to 0.444) | 58.6% |
|  | AOM | 683.7±39.1 | 353.7 | 0.52 (0.494 to 0.546) | 48.0% |
|  | Asthma | 62.4±3.5 | 32.7 | 0.531 (0.447 to 0.63) | 46.9% |
| AGE visits^˧^ |  | 459.1±23.5 | 249.2 | 0.545 (0.513 to 0.579) | 45.5% |
| Non-respiratory, non-AGE visits^˧^ |  | 836.6±29.4 | 578.4 | 0.87 (0.821 to 0.922) | 13.0% |
|  | UTI | 33±1.1 | 27.6 | 0.834 (0.679 to 1.024) | 16.6% |
|  | Epilepsy | 19.3±0.4 | 16.6 | 0.855 (0.656 to 1.114) | 14.5% |
|  | Trauma | 244.8±5.9 | 221.6 | 0.905 (0.842 to 0.972) | 9.5% |
|  | SSTI | 79.6±1.9 | 62.8 | 0.779 (0.682 to 0.89) | 22.1% |
|  |  |  |  |  |  |
|  |  |  | Prescriptions |  |  |
|  |  |  |  |  |  |
| All respiratory prescriptions^˧^ |  | 2808±183.7 | 1443.4 | 0.526 (0.514 to 0.538) | 47.4% |
|  |  |  |  |  |  |
| Respiratory antibiotics prescriptions^˧^ |  | 1341.3±110.1 | 660.6 | 0.504 (0.486 to 0.523) | 49.6% |
|  | Amoxicillin/  Amoxicillin clavulanate | 1050.1±83.6 | 509.3 | 0.496 (0.476 to 0.517) | 50.4% |
|  | Azithromycin | 266.7±22.7 | 140.4 | 0.54 (0.498 to 0.585) | 46.0% |
|  | Ceftriaxone | 24.5±3.8 | 10.8 | 0.438 (0.324 to 0.592) | 56.2% |
| Respiratory non-antibiotic prescriptions^˧^ |  | 1466.7±74.2 | 782.8 | 0.546 (0.528 to 0.565) | 45.4% |
|  | Asthma inhalators and solutions | 598.9±14.5 | 331.8 | 0.568 (0.538 to 0.598) | 43.2% |
|  | Throat relief and nasal congestion | 867.9±68.2 | 451 | 0.531 (0.508 to 0.555) | 46.9% |
| All non-respiratory prescriptions^˧^ |  | 115.6±5.6 | 103.7 | 0.901 (0.81 to 1.002) | 9.9% |
|  |  |  |  |  |  |
| Non-respiratory antibiotics prescriptions^˧^ |  | 88±5.5 | 76.5 | 0.862 (0.763 to 0.974) | 13.8% |
|  | OFGC | 73.4±5.8 | 65.6 | 0.889 (0.778 to 1.015) | 11.1% |
|  | TMP/SMX | 14.6±1.1 | 10.8 | 0.73 (0.534 to 0.998) | 27.0% |
| Non-respiratory, non-antibiotics prescriptions^˧^ | Anti-epileptic | 27.5±0.4 | 27.2 | 1.032 (0.832 to 1.281) | -3.2% |
| \| URI, upper respiratory tract infection; LRI, lower respiratory tract infection; AOM, acute otitis media; AGE, acute gastroenteritis; UTI, urinary tract infection; SSTI, skin and soft tissue infection; OFGC, oral first generation cephalosporins; TMP/SMX, trimethoprim/sulfamethoxazole  ^˧^per 1,000 children \| \| --- \| | | | | | |

**Online Resource 1.6: Yearly rates and rate ratios 2020-2021 vs. 2016-2019 in Jewish children < 5 years old**

|  |  | Mean yearly rate  Feb 2016-Jan 2020 | Yearly rate  Feb 2020-Jan 2021 | Rate Ratio | Reduction |
| --- | --- | --- | --- | --- | --- |
|  |  |  |  |  |  |
|  |  |  | Visits |  |  |
|  |  |  |  |  |  |
| Overall clinic visits^˧^ |  | 8950.6±170.7 | 6987.3 | 0.781 (0.778 to 0.784) | 21.9% |
| Respiratory visits^˧^ |  | 4299.8±170.3 | 2145.5 | 0.500 (0.496 to 0.503) | 50.0% |
|  | URI | 3024.1±120.3 | 1563.2 | 0.517 (0.513 to 0.522) | 48.3% |
|  | LRI | 452.0±15.3 | 165.1 | 0.366 (0.356 to 0.375) | 63.4% |
|  | AOM | 739.5±33.8 | 374.6 | 0.507 (0.498 to 0.516) | 49.3% |
|  | Asthma | 84.2±8.4 | 42.6 | 0.507 (0.482 to 0.534) | 49.3% |
| AGE visits^˧^ |  | 461.3±39.7 | 253.6 | 0.551 (0.539 to 0.563) | 44.9% |
| Non-respiratory, non-AGE visits^˧^ |  | 408.9±11.9 | 392.2 | 0.960 (0.943 to 0.977) | 4.0% |
|  | UTI | 40.6±1.2 | 35.3 | 0.870 (0.820 to 0.923) | 13.0% |
|  | Epilepsy | 20.5±0.9 | 18.5 | 0.903 (0.832 to 0.980) | 9.7% |
|  | Trauma | 270.5±9.1 | 271.7 | 1.005 (0.984 to 1.027) | -0.5% |
|  | SSTI | 77.3±2.3 | 66.6 | 0.862 (0.826 to 0.900) | 13.8% |
|  |  |  |  |  |  |
|  |  |  | Prescriptions |  |  |
|  |  |  |  |  |  |
| All respiratory prescriptions^˧^ |  | 3176.4±137.6 | 1642.9 | 0.518 (0.514 to 0.522) | 48.2% |
|  |  |  |  |  |  |
| Respiratory antibiotics prescriptions^˧^ |  | 1344.3±77.1 | 629.8 | 0.469 (0.463 to 0.476) | 53.1% |
|  | Amoxicillin/  Amoxicillin clavulanate | 1047.5±49.5 | 488.6 | 0.467 (0.460 to 0.474) | 53.3% |
|  | Azithromycin | 278.7±26.3 | 134.5 | 0.484 (0.470 to 0.498) | 51.6% |
|  | Ceftriaxone | 18.2±1.9 | 6.7 | 0.372 (0.327 to 0.422) | 62.8% |
| Respiratory non-antibiotic prescriptions^˧^ |  | 1832.1±61.2 | 1013.1 | 0.553 (0.548 to 0.559) | 44.7% |
|  | Asthma inhalators and solutions | 868.9±17.2 | 483.1 | 0.556 (0.547 to 0.564) | 44.4% |
|  | Throat relief and nasal congestion | 963.2±67.7 | 530.0 | 0.551 (0.543 to 0.560) | 44.9% |
| All non-respiratory prescriptions^˧^ |  | 115.7±7.1 | 116.1 | 1.002 (0.969 to 1.035) | -0.2% |
|  |  |  |  |  |  |
| Non-respiratory antibiotics prescriptions^˧^ |  | 92.9±4.2 | 88.6 | 0.953 (0.918 to 0.989) | 4.7% |
|  | OFGC | 80.1±4.7 | 78.2 | 0.975 (0.936 to 1.015) | 2.5% |
|  | TMP/SMX | 12.8±0.7 | 10.4 | 0.816 (0.733 to 0.909) | 18.4% |
| Non-respiratory, non-antibiotics prescriptions^˧^ | Anti-epileptic | 22.8±3.1 | 27.4 | 1.200 (1.119 to 1.286) | -20.0% |
| URI, upper respiratory tract infection; LRI, lower respiratory tract infection; AOM, acute otitis media; AGE, acute gastroenteritis; UTI, urinary tract infection; SSTI, skin and soft tissue infection; OFGC, oral first generation cephalosporins; TMP/SMX, trimethoprim/sulfamethoxazole  ^˧^per 1,000 children | | | | | |

**Online Resource 1.7: Yearly rates and rate ratios 2020-2021 vs. 2016-2019 in Bedouin children < 5 years old**

|  |  | Mean yearly rate  Feb 2016-Jan 2020 | Yearly rate  Feb 2020-Jan 2021 | Rate Ratio | Reduction |
| --- | --- | --- | --- | --- | --- |
|  |  |  |  |  |  |
|  |  |  | Visits |  |  |
|  |  |  |  |  |  |
| Overall clinic visits^˧^ |  | 6310.8±291.9 | 4199.1 | 0.666 (0.663 to 0.67) | 33.4% |
| Respiratory visits^˧^ |  | 3424.1±253.9 | 1680.4 | 0.492 (0.488 to 0.496) | 50.8% |
|  | URI | 2325.9±178.5 | 1115.4 | 0.481 (0.476 to 0.486) | 51.9% |
|  | LRI | 385.5±24.6 | 183.2 | 0.476 (0.464 to 0.488) | 52.4% |
|  | AOM | 673.3±61.1 | 358.7 | 0.534 (0.524 to 0.544) | 46.6% |
|  | Asthma | 39.5±0.8 | 23.1 | 0.586 (0.544 to 0.631) | 41.4% |
| AGE visits^˧^ |  | 489.3±33.9 | 263.5 | 0.539 (0.527 to 0.551) | 46.1% |
| Non-respiratory, non-AGE visits^˧^ |  | 868.3±45.4 | 552.4 | 0.637 (0.627 to 0.646) | 36.3% |
|  | UTI | 25.7±2.4 | 19.7 | 0.771 (0.711 to 0.837) | 22.9% |
|  | Epilepsy | 19.1±0.8 | 15.3 | 0.798 (0.727 to 0.876) | 20.2% |
|  | Trauma | 244.3±7.7 | 191 | 0.783 (0.762 to 0.804) | 21.7% |
|  | SSTI | 89.2±3.7 | 62.4 | 0.7 (0.669 to 0.733) | 30.0% |
|  |  |  |  |  |  |
|  |  |  | Prescriptions |  |  |
|  |  |  |  |  |  |
| All respiratory prescriptions^˧^ |  | 2482.8±235.7 | 1330.4 | 0.538 (0.532 to 0.543) | 46.2% |
|  |  |  |  |  |  |
| Respiratory antibiotics prescriptions^˧^ |  | 1383.5±152.8 | 746.4 | 0.541 (0.534 to 0.548) | 45.9% |
|  | Amoxicillin/  Amoxicillin clavulanate | 1078.4±123.9 | 566.8 | 0.528 (0.52 to 0.535) | 47.2% |
|  | Azithromycin | 276.7±26.4 | 166 | 0.601 (0.585 to 0.618) | 39.9% |
|  | Ceftriaxone | 28.4±5.8 | 13.7 | 0.484 (0.44 to 0.532) | 51.6% |
| Respiratory non-antibiotic prescriptions^˧^ |  | 1099.3±83.8 | 584 | 0.533 (0.525 to 0.54) | 46.7% |
|  | Asthma inhalators and solutions | 318.2±17.9 | 191.8 | 0.603 (0.588 to 0.619) | 39.7% |
|  | Throat relief and nasal congestion | 781.1±68.8 | 392.2 | 0.504 (0.495 to 0.513) | 49.6% |
| All non-respiratory prescriptions^˧^ |  | 123.7±9.5 | 98.5 | 0.797 (0.768 to 0.827) | 20.3% |
|  |  |  |  |  |  |
| Non-respiratory antibiotics prescriptions^˧^ |  | 91.8±9 | 69.9 | 0.761 (0.729 to 0.795) | 23.9% |
|  | OFGC | 73.2±8.4 | 57.6 | 0.786 (0.749 to 0.824) | 21.4% |
|  | TMP/SMX | 18.6±2.9 | 12.3 | 0.665 (0.6 to 0.736) | 33.5% |
| Non-respiratory, non-antibiotics prescriptions^˧^ | Anti-epileptic | 31.9±4.3 | 28.6 | 0.9 (0.84 to 0.964) | 10.0% |

URI, upper respiratory tract infection; LRI, lower respiratory tract infection; AOM, acute otitis media; AGE, acute gastroenteritis; UTI, urinary tract infection; SSTI, skin and soft tissue infection; OFGC, oral first generation cephalosporins; TMP/SMX, trimethoprim/sulfamethoxazole

^˧^per 1,000 children

**Online Resource 1.8: Yearly rates and rate ratios 2020-2021 vs. 2016-2019, adjusted for ethnicity in children 5-17 years old**

|  |  | Mean yearly rate  Feb 2016-Jan 2020 | Yearly rate  Feb 2020-Jan 2021 | Rate Ratio* | Reduction* |
| --- | --- | --- | --- | --- | --- |
|  |  |  |  |  |  |
|  |  |  | Visits |  |  |
|  |  |  |  |  |  |
| Overall clinic visits^˧^ |  | 3511.1±47.6 | 2948.9 | 0.831 (0.822 to 0.841) | 16.9% |
| Respiratory visits^˧^ |  | 994.5±36.8 | 516.8 | 0.519 (0.504 to 0.534) | 48.1% |
|  | URI | 848.9±30.2 | 430 | 0.506 (0.49 to 0.522) | 49.4% |
|  | LRI | 34.8±2.8 | 17.7 | 0.516 (0.439 to 0.606) | 48.4% |
|  | AOM | 82.6±3.2 | 49.3 | 0.591 (0.535 to 0.652) | 40.9% |
|  | Asthma | 28.2±1.6 | 19.8 | 0.703 (0.597 to 0.828) | 29.7% |
| AGE visits^˧^ |  | 74.2±5.9 | 35.5 | 0.48 (0.428 to 0.537) | 52.0% |
| Non-respiratory, non-AGE visits^˧^ |  | 394.8±16.5 | 284.7 | 0.771 (0.736 to 0.807) | 22.9% |
|  | UTI | 18.3±0.8 | 14.6 | 0.798 (0.658 to 0.968) | 20.2% |
|  | Epilepsy | 14.1±1.3 | 17.4 | 1.213 (0.993 to 1.481) | -21.3% |
|  | Trauma | 240.8±10.9 | 180.2 | 0.746 (0.707 to 0.787) | 25.4% |
|  | SSTI | 46.7±1.2 | 36.2 | 0.759 (0.672 to 0.858) | 24.1% |
|  |  |  |  |  |  |
|  |  |  | Prescriptions |  |  |
|  |  |  |  |  |  |
| All respiratory prescriptions^˧^ |  | 970.5±50.3 | 540.4 | 0.559 (0.543 to 0.575) | 44.1% |
|  |  |  |  |  |  |
| Respiratory antibiotics prescriptions^˧^ |  | 483±26.4 | 253.6 | 0.529 (0.507 to 0.552) | 47.1% |
|  | Amoxicillin/  Amoxicillin clavulanate | 421.4±25.5 | 221.8 | 0.53 (0.506 to 0.554) | 47.0% |
|  | Azithromycin | 56.8±1.7 | 29.6 | 0.525 (0.463 to 0.596) | 47.5% |
|  | Ceftriaxone | 4.8±0.5 | 2.3 | 0.524 (0.33 to 0.831) | 47.6% |
| Respiratory non-antibiotic prescriptions^˧^ |  | 487.5±24.8 | 286.7 | 0.588 (0.565 to 0.613) | 41.2% |
|  | Asthma inhalators and solutions | 108.9±3.2 | 82.6 | 0.76 (0.701 to 0.824) | 24.0% |
|  | Throat relief and nasal congestion | 378.6±22.2 | 204.1 | 0.538 (0.513 to 0.565) | 46.2% |
| All non-respiratory prescriptions^˧^ |  | 120.1±2.7 | 113.2 | 0.927 (0.862 to 0.997) | 7.3% |
|  |  |  |  |  |  |
| Non-respiratory antibiotics prescriptions^˧^ |  | 40.4±1.7 | 33.6 | 0.812 (0.714 to 0.924) | 18.8% |
|  | OFGC | 34.8±1.9 | 30 | 0.84 (0.732 to 0.964) | 16.0% |
|  | TMP/SMX | 5.6±0.6 | 3.6 | 0.642 (0.444 to 0.929) | 35.8% |
| Non-respiratory, non-antibiotics prescriptions^˧^ | Anti-epileptic | 79.7±1.4 | 79.7 | 0.987 (0.903 to 1.078) | 1.3% |
| *adjusted for ethnicity  ^˧^per 1,000 children  URI, upper respiratory tract infection; LRI, lower respiratory tract infection; AOM, acute otitis media; AGE, acute gastroenteritis; UTI, urinary tract infection; SSTI, skin and soft tissue infection; OFGC, oral first generation cephalosporins; TMP/SMX, trimethoprim/sulfamethoxazole | | | | | |

**Online Resource 1.9: Yearly rates and rate ratios 2020-2021 vs. 2016-2019 in Jewish children 5-17 years old**

|  |  | Mean yearly rate  Feb 2016-Jan 2020 | Yearly rate  Feb 2020-Jan 2021 | Rate Ratio | Reduction |
| --- | --- | --- | --- | --- | --- |
|  |  |  |  |  |  |
|  |  |  | Visits |  |  |
|  |  |  |  |  |  |
| Overall clinic visits^˧^ |  | 4328.4±36 | 3863.5 | 0.893 (0.889 to 0.896) | 10.7% |
| Respiratory visits^˧^ |  | 1147.5±34.6 | 620.2 | 0.541 (0.536 to 0.546) | 45.9% |
|  | URI | 991.6±28.6 | 521.9 | 0.527 (0.522 to 0.532) | 47.3% |
|  | LRI | 34.5±1.9 | 18.1 | 0.526 (0.498 to 0.556) | 47.4% |
|  | AOM | 93.4±4.7 | 57.3 | 0.615 (0.596 to 0.634) | 38.5% |
|  | Asthma | 28.1±1.3 | 22.9 | 0.818 (0.778 to 0.86) | 18.2% |
| AGE visits^˧^ |  | 95.3±11 | 43.9 | 0.463 (0.447 to 0.479) | 53.7% |
| Non-respiratory, non-AGE visits^˧^ |  | 433.2±25.6 | 318.7 | 0.737 (0.727 to 0.747) | 26.3% |
|  | UTI | 20±1.2 | 16.9 | 0.844 (0.796 to 0.895) | 15.6% |
|  | Epilepsy | 14.4±1.1 | 19.7 | 1.36 (1.285 to 1.441) | -36.0% |
|  | Trauma | 258.1±13.8 | 200.9 | 0.78 (0.767 to 0.793) | 22.0% |
|  | SSTI | 45±1.8 | 37 | 0.823 (0.791 to 0.856) | 17.7% |
|  |  |  |  |  |  |
|  |  |  | Prescriptions |  |  |
|  |  |  |  |  |  |
| All respiratory prescriptions^˧^ |  | 1075.7±34 | 608.5 | 0.566 (0.561 to 0.572) | 43.4% |
|  |  |  |  |  |  |
| Respiratory antibiotics prescriptions^˧^ |  | 509.2±11 | 271.6 | 0.534 (0.526 to 0.541) | 46.6% |
|  | Amoxicillin/  Amoxicillin clavulanate | 436.2±12.9 | 235.8 | 0.541 (0.533 to 0.549) | 45.9% |
|  | Azithromycin | 70.4±3.1 | 34.8 | 0.493 (0.475 to 0.513) | 50.7% |
|  | Ceftriaxone | 2.6±0.2 | 1 | 0.388 (0.309 to 0.486) | 61.2% |
| Respiratory non-antibiotic prescriptions^˧^ |  | 566.5±24.3 | 336.9 | 0.596 (0.588 to 0.603) | 40.4% |
|  | Asthma inhalators and solutions | 137±3.6 | 106.6 | 0.778 (0.76 to 0.796) | 22.2% |
|  | Throat relief and nasal congestion | 429.5±25.3 | 230.3 | 0.538 (0.529 to 0.546) | 46.2% |
| All non-respiratory prescriptions^˧^ |  | 124.3±3.7 | 114.6 | 0.923 (0.903 to 0.944) | 7.7% |
|  |  |  |  |  |  |
| Non-respiratory antibiotics prescriptions^˧^ |  | 43.7±0.5 | 39 | 0.893 (0.859 to 0.928) | 10.7% |
|  | OFGC | 38.6±1 | 35.4 | 0.915 (0.878 to 0.953) | 8.5% |
|  | TMP/SMX | 5±0.6 | 3.6 | 0.721 (0.637 to 0.817) | 27.9% |
| Non-respiratory, non-antibiotics prescriptions^˧^ | Anti-epileptic | 80.6±4.2 | 75.6 | 0.94 (0.914 to 0.967) | 6.0% |
| URI, upper respiratory tract infection; LRI, lower respiratory tract infection; AOM, acute otitis media; AGE, acute gastroenteritis; UTI, urinary tract infection; SSTI, skin and soft tissue infection; OFGC, oral first generation cephalosporins; TMP/SMX, trimethoprim/sulfamethoxazole  ^˧^per 1,000 children | | | | | |

**Online Resource 1.10: Yearly rates and rate ratios 2020-2021 vs. 2016-2019; Bedouin children 5-17y**

|  |  | Mean yearly rate  Feb 2016-Jan 2020 | Yearly rate  Feb 2020-Jan 2021 | Rate Ratio | Reduction |
| --- | --- | --- | --- | --- | --- |
|  |  |  |  |  |  |
|  |  |  | Visits |  |  |
|  |  |  |  |  |  |
| Overall clinic visits^˧^ |  | 2546.1±84.3 | 1832.6 | 0.72 (0.716 to 0.724) | 28.0% |
| Respiratory visits^˧^ |  | 780.2±44 | 377.2 | 0.484 (0.478 to 0.49) | 51.6% |
|  | URI | 648.2±36.1 | 305 | 0.471 (0.464 to 0.477) | 52.9% |
|  | LRI | 33.2±4.4 | 16.7 | 0.504 (0.475 to 0.535) | 49.6% |
|  | AOM | 71.7±2.9 | 40 | 0.557 (0.536 to 0.579) | 44.3% |
|  | Asthma | 27.1±1.9 | 15.6 | 0.576 (0.542 to 0.613) | 42.4% |
| AGE visits^˧^ |  | 48.8±2 | 25.1 | 0.515 (0.49 to 0.54) | 48.5% |
| Non-respiratory, non-AGE visits^˧^ |  | 360±14.2 | 249.7 | 0.694 (0.683 to 0.705) | 30.6% |
|  | UTI | 16.5±1 | 12.2 | 0.738 (0.687 to 0.793) | 26.2% |
|  | Epilepsy | 13.5±1.3 | 14.1 | 1.044 (0.975 to 1.117) | -4.4% |
|  | Trauma | 230.8±11.3 | 162.6 | 0.705 (0.691 to 0.719) | 29.5% |
|  | SSTI | 49.5±2 | 34.5 | 0.698 (0.669 to 0.728) | 30.2% |
|  |  |  |  |  |  |
|  |  |  | Prescriptions |  |  |
|  |  |  |  |  |  |
| All respiratory prescriptions^˧^ |  | 805.8±66.3 | 440.7 | 0.547 (0.541 to 0.554) | 45.3% |
|  |  |  |  |  |  |
| Respiratory antibiotics prescriptions^˧^ |  | 436±40.5 | 227.9 | 0.523 (0.515 to 0.532) | 47.7% |
|  | Amoxicillin/  Amoxicillin clavulanate | 392.5±36.7 | 202.4 | 0.516 (0.507 to 0.525) | 48.4% |
|  | Azithromycin | 37.7±3.1 | 22.2 | 0.588 (0.559 to 0.62) | 41.2% |
|  | Ceftriaxone | 5.8±1 | 3.4 | 0.59 (0.517 to 0.674) | 41.0% |
| Respiratory non-antibiotic prescriptions^˧^ |  | 369.8±26.1 | 212.8 | 0.576 (0.566 to 0.585) | 42.4% |
|  | Asthma inhalators and solutions | 72.1±7.1 | 52.2 | 0.724 (0.7 to 0.749) | 27.6% |
|  | Throat relief and nasal congestion | 297.6±19.2 | 160.6 | 0.54 (0.53 to 0.55) | 46.0% |
| All non-respiratory prescriptions^˧^ |  | 113.4±5.4 | 105.7 | 0.931 (0.909 to 0.955) | 6.9% |
|  |  |  |  |  |  |
| Non-respiratory antibiotics prescriptions^˧^ |  | 37.3±3.2 | 26.6 | 0.712 (0.678 to 0.747) | 28.8% |
|  | OFGC | 30.9±2.9 | 22.9 | 0.74 (0.703 to 0.779) | 26.0% |
|  | TMP/SMX | 6.4±0.8 | 3.7 | 0.577 (0.508 to 0.654) | 42.3% |
| Non-respiratory, non-antibiotics prescriptions^˧^ | Anti-epileptic | 76.1±2.7 | 79.1 | 1.039 (1.01 to 1.07) | -3.9% |
| URI, upper respiratory tract infection; LRI, lower respiratory tract infection; AOM, acute otitis media; AGE, acute gastroenteritis; UTI, urinary tract infection; SSTI, skin and soft tissue infection; OFGC, oral first generation cephalosporins; TMP/SMX, trimethoprim/sulfamethoxazole  ^˧^per 1,000 children | | | | | |
