# Supplementary material for: Disproportionate reduction in respiratory vs. non-respiratory outpatient clinic visits and antibiotic use in children during the COVID-19 pandemic
Source: BMC Pediatr. 2022 May 6;22:254. doi: 10.1186/s12887-022-03315-0 (PMC9073498; doi:10.1186/s12887-022-03315-0)
Supplement: Supplementary file 2 — Additional file 2. [file 12887_2022_3315_MOESM2_ESM.docx]

**ONLINE RESOURCE 2: SUPPLEMENTARY FIGURES**

**Disproportionate Reduction in Respiratory Vs. Non-Respiratory Outpatient Clinic Visits and Antibiotic Use in Children During the COVID-19 Pandemic**

**Noga Givon-Lavi*,PhD^1, 2^ Dana Danino*, MD^1, 2^ Bart Adriaan Van Der Beek,^1^ Amir Sharf,^3^ David Greenberg, MD^1,2^ Shalom Ben-Shimol, MD^1, 2^**

^1^ Faculty of Health Sciences, Ben-Gurion University of the Negev, Beer-Sheva, Israel

^2^ The Pediatric Infectious Disease Unit, Soroka University Medical Center, Beer-Sheva, Israel

^3^Economics and Data Analysis Department, Clalit HMO South district, Beer-Sheva, Israel

*Both authors contributed equally to this manuscript

**Corresponding author:**

Dana Danino, MD

The Pediatric Infectious Disease Unit

Soroka University Medical Center

Beer-Sheva, Israel.

E-mail: danadanino@hotmail.com

Tel: 972-8-6400547

Fax: 972-8-6232334

**Online Resource 2.1:** Monthly rates of URI, LRI, AOM and asthma outpatient clinical visits in the pre-COVID-19 (2016-2019) and the COVID-19 period.


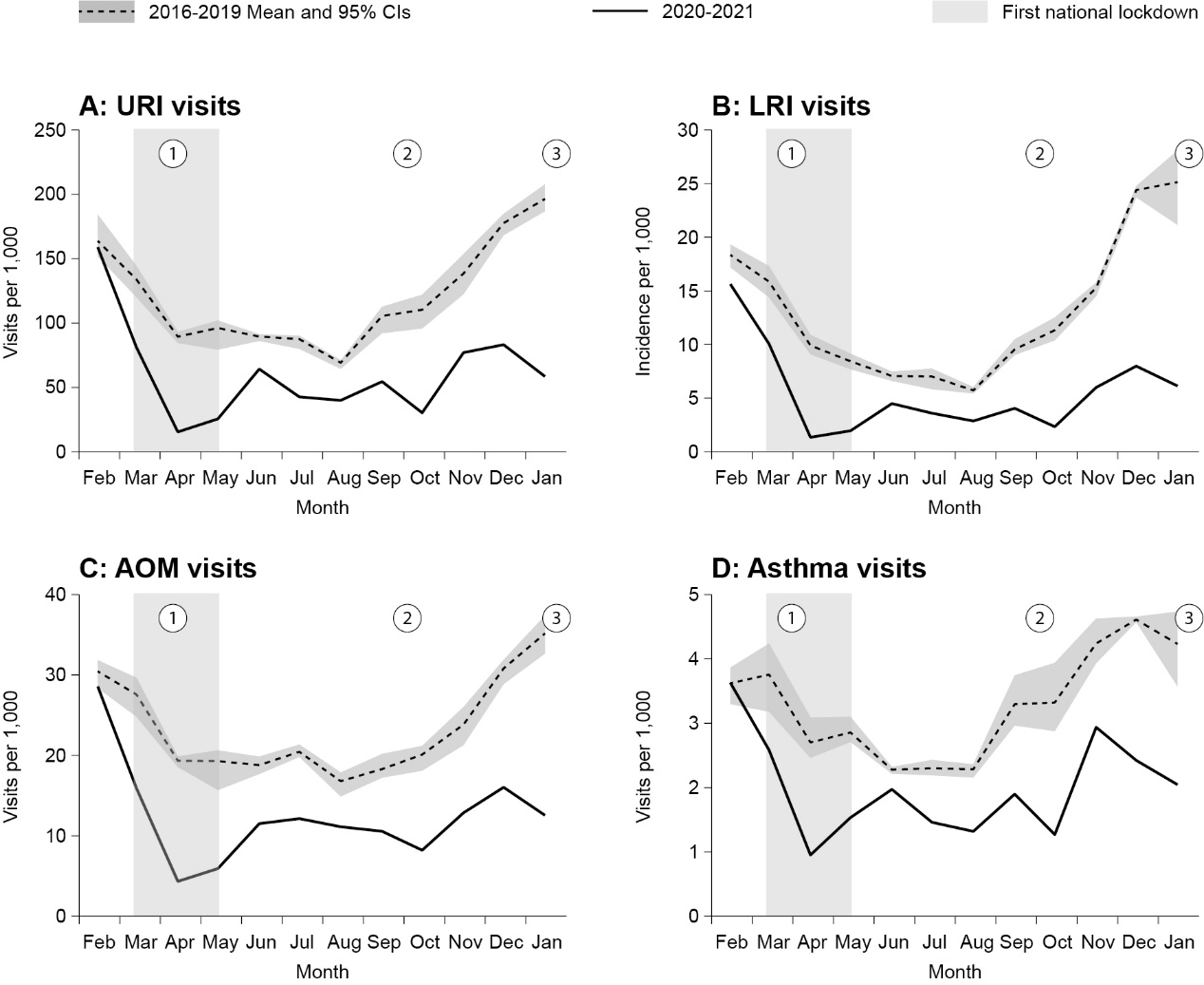


**Online Resource 2.2:** Monthly rates of UTI, epilepsy, SSTI and trauma outpatient clinical visits in the pre-COVID-19 (2016-2019) and the COVID-19 period.


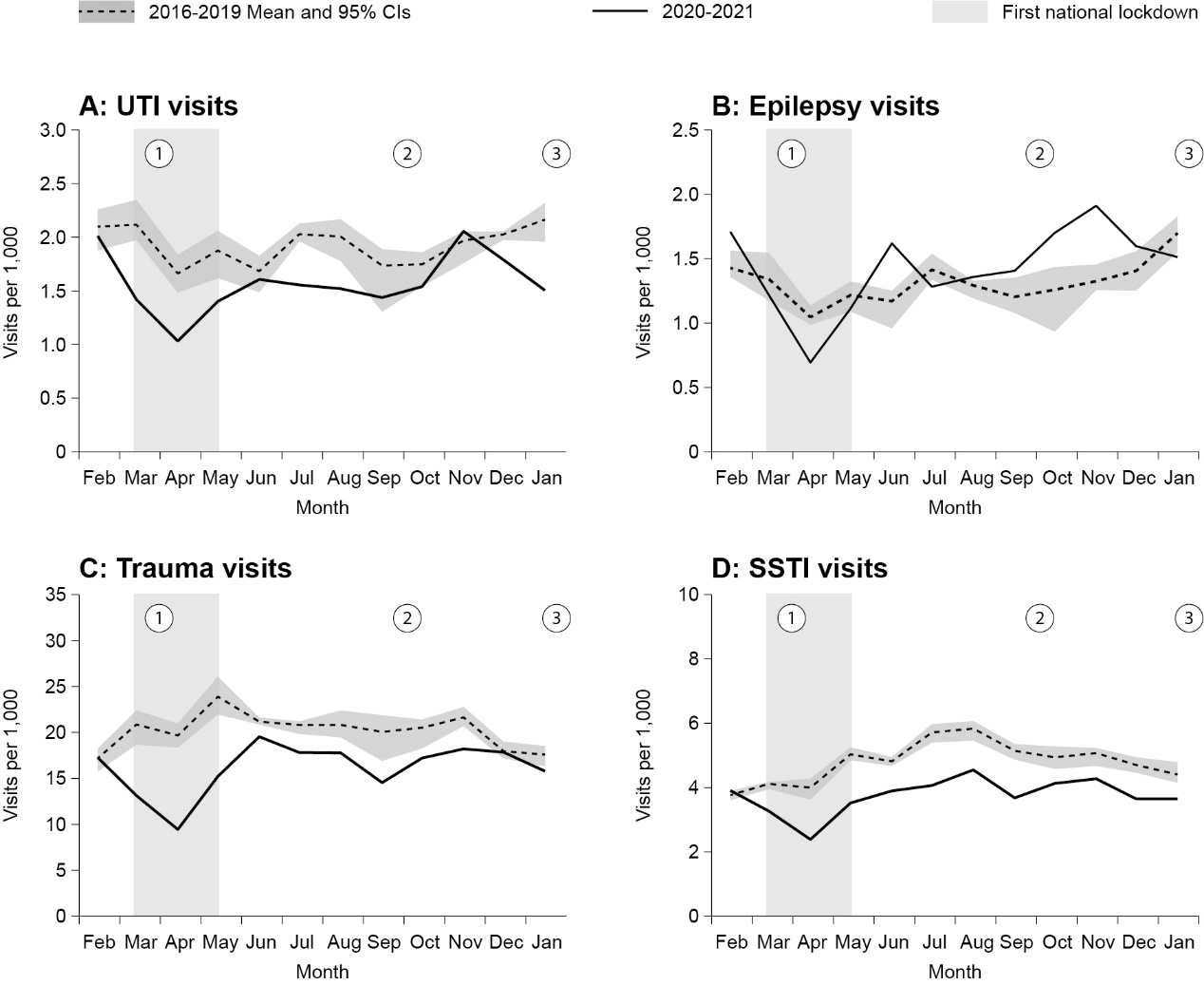


**Online Resource 2.3:** Monthly rates of respiratory antibiotics dispensed prescriptions in the pre-COVID-19 (2016-2019) and the COVID-19 period.


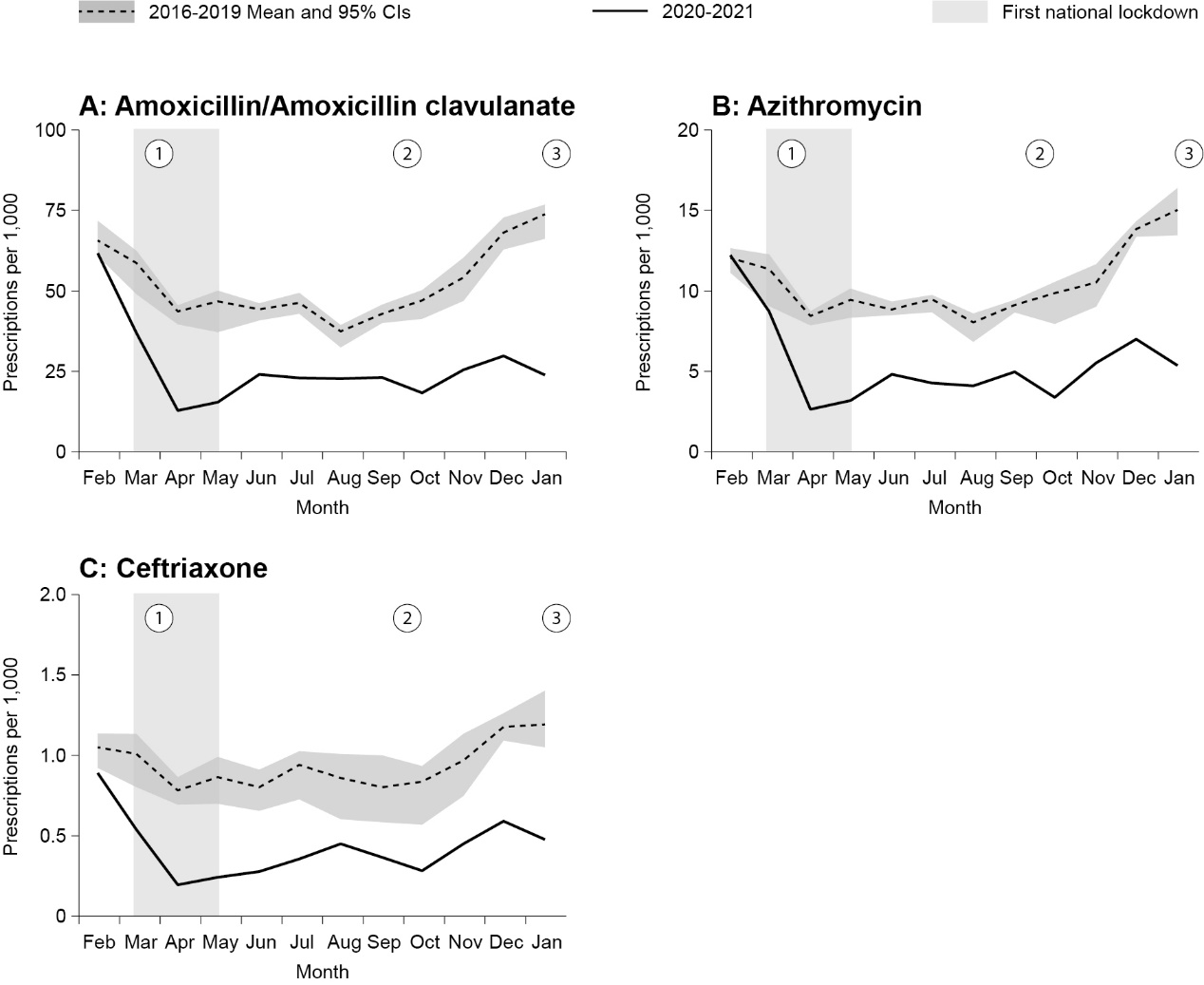


**Online Resource 2.4:** Monthly rates of non-antibiotics respiratory medications prescriptions in the pre-COVID-19 (2016-2019) and the COVID-19 period.

**
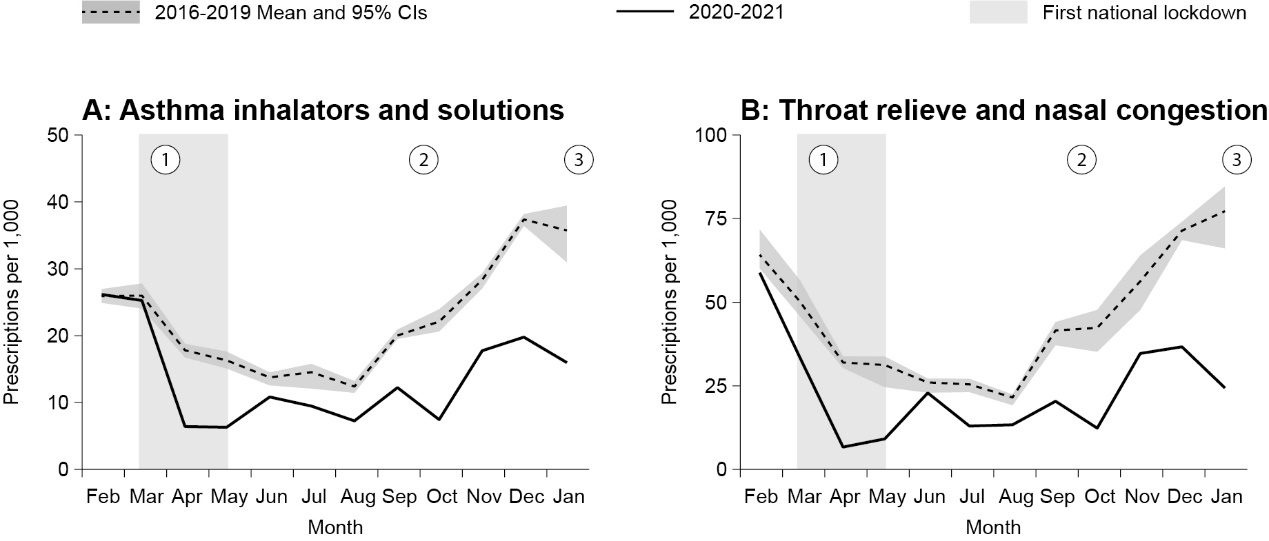
**

**Online Resource 2.5:** Monthly rates of non-respiratory antibiotics dispensed prescriptions in the pre-COVID-19 (2016-2019) and the COVID-19 period.

**
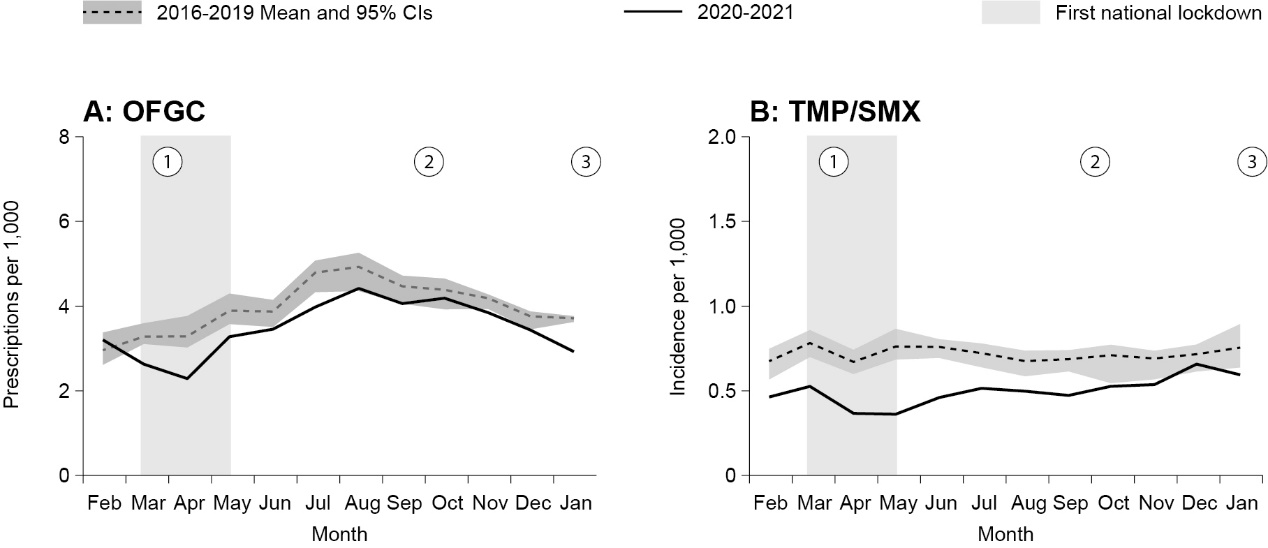
**
